# Molecular Force Sensors for Biological Application

**DOI:** 10.3390/ijms25116198

**Published:** 2024-06-04

**Authors:** Huiyan Chen, Shouhan Wang, Yi Cao, Hai Lei

**Affiliations:** 1 National Laboratory of Solid State Microstructure, Department of Physics, Nanjing University, Nanjing 210093, China; dg20220003@smail.nju.edu.cn (H.C.); mg21220203@smail.nju.edu.cn (S.W.); 2 School of Physics, Zhejiang University, Hangzhou 310027, China; 3 Institute for Advanced Study in Physics, Zhejiang University, Hangzhou 310027, China

**Keywords:** cellular traction forces, traction force microscopy, fluorescent molecular force sensors, mechanotransduction

## Abstract

The mechanical forces exerted by cells on their surrounding microenvironment are known as cellular traction forces. These forces play crucial roles in various biological processes, such as tissue development, wound healing and cell functions. However, it is hard for traditional techniques to measure cellular traction forces accurately because their magnitude (from pN to nN) and the length scales over which they occur (from nm to μm) are extremely small. In order to fully understand mechanotransduction, highly sensitive tools for measuring cellular forces are needed. Current powerful techniques for measuring traction forces include traction force microscopy (TFM) and fluorescent molecular force sensors (FMFS). In this review, we elucidate the force imaging principles of TFM and FMFS. Then we highlight the application of FMFS in a variety of biological processes and offer our perspectives and insights into the potential applications of FMFS.

## 1. Introduction

The majority of cells in vivo are subjected to a range of complex mechanical forces. For example, vascular smooth muscle cells suffer from cyclic stretch forces due to the rhythmic expansion of the arterial wall [1]. Vascular endothelial cells are exposed to the fluid shear stress of blood flow [2]. In recent years, an increasing body of research has shown that mechanical forces play a significant regulatory role in cell behaviors and functions such as adhesion [3,4,5], migration [6,7,8,9], proliferation [10,11], and differentiation [12,13,14,15]. The maintenance of cell functions relies on cell–matrix interactions and cellular mechanotransduction. During this important biological process, cells exert, sense and respond to mechanical forces via cell–extracellular matrix (ECM) adhesions and cell–cell adhesions [16,17]. Specifically, external mechanical forces are transmitted into cells through mechanosensors, trigger intracellular biochemical signal cascades and change gene expression to mediate cellular behaviors [18]. Concurrently, cells respond to external mechanical forces in a wide range of ways, such as adjusting traction forces or secreting extracellular proteins. For instance, during wound healing, fibroblasts remodel the collagen matrix by applying traction forces [19]. In stiffened tumors, cells secrete high levels of matrix metalloproteinases to remodel their surrounding matrix and create migration space [20]. Anomalies in cellular mechanosensing capabilities would lead to the occurrence of diseases such as muscular atrophy [21,22], cancer [23], and vascular diseases [24,25].

Mechanotransduction involves interactions between protein molecules and protein conformational changes induced by pN mechanical forces [26,27,28]. Therefore, the development of tools to quantify cellular pN forces is crucial for fully understanding of molecules mechanotransduction. The current technologies used for quantifying cellular forces include single-molecule force spectroscopy (such as atomic force microscopy [29], optical tweezers [30], and magnetic tweezers [31]), elastic resonator interference stress microscopy, photonic crystal cellular force microscopy, TFM and FMFS. Athough single-molecule force spectroscopy techniques can measure pN forces, these techniques actively apply force to cells and cannot obtain images of cellular forces [32]. In contrast, TFM and FMFS passively detect the mechanical forces generated by cells and provide images of the force distribution. TFM maps force information by measuring the substrate deformation generated by cellular forces (i.e., converting forces into strain), whereas FMFS reports the magnitude and distribution of cellular forces through force-sensitive fluorescent reporter systems (i.e., converting forces into fluorescence intensity changes). In this review, we elucidate the measurement and imaging principles of TFM and FMFS, and we are particularly focused on the application of FMFS, such as cell–ECM or cell–cell interactions, monitoring and regulation of cellular behaviors, and high-throughput platforms. Finally, we discuss the future developments and potential applications of FMFS.

## 2. Traction Force Microscope

In biological processes such as spreading and migration, cells exert mechanical forces on the extracellular matrix (ECM) and induce the deformation of substrate materials. In 1980, Harris et al. [33] first discovered that the wrinkling of ultrathin silicone rubber can be used to estimate cellular traction forces during cell crawling. This pioneering work laid the foundation for the use of TFM to measure cellular forces. To obtain substrates with variable physical properties, Pelham and Wang introduced polyacrylamide hydrogels as substrates [3]. The stiffness of elastic substrates is determined by the concentration of acrylamide and bis-acrylamide, which makes it possible to quantify cell forces. To visualize the deformation of substrates easily, Dembo et al. [34] embedded fluorescent microspheres randomly throughout the polyacrylamide hydrogel. Based on the displacement of these microspheres, cell forces exerted on the substrate can be reconstructed. Methods with key markers such as fluorescent microbeads or micropillar arrays for mapping cellular forces further promoted the development of TFM. Since then, TFM has continuously developed and become one of the standard tools for measuring cellular traction forces [35,36,37,38,39,40].

By precisely tracking marker positions and measuring substrate deformation, the cellular traction force field can be reconstructed. In addition, natural ordered biopolymers such as collagen can be used in TFM as natural markers, because of their inherent optical properties of birefringence [41,42,43]. For instance, Laforgue et al. [42] used collagen as 3D substrates and natural markers. By following collagen fiber deformations, they determined the 3D displacements field induced by cancer cell migration. These kinds of 3D natural matrices are similar to the native cellular microenvironment and can be used for real-time measurement. However, due to the narrow linear elastic range and nonlinear mechanical properties of fibrous natural polymers, reconstructing force fields from displacement fields presents significant challenges and complexities. These methods usually provide qualitative tension maps of fiber network deformation rather than quantitative force maps. With the ongoing optimization of algorithms, these materials have great potential for measuring cell forces. Based on elasticity theory and finite element analysis, the cell traction forces within the target area can be reconstructed from the displacement of markers [37]. Because the experiments rely on microscopic imaging, the substrate materials (such as polyacrylamide and polydimethylsiloxane) used for TFM need to possess good optical transparency [44].

### 2.1. Two Dimensional Traction Force Microscope Based on Flat Elastic Substrates

**Figure 1:**
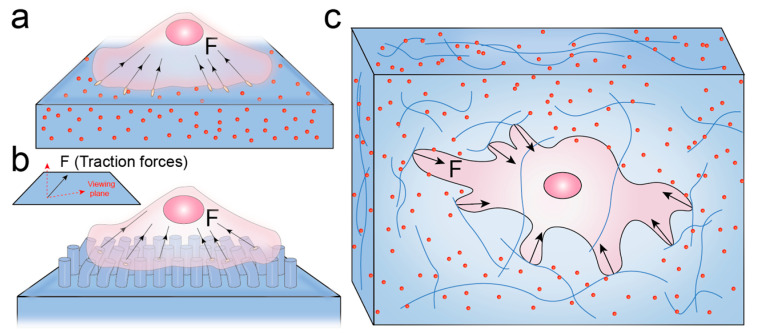
Overview of TFM used to quantify cell traction forces (F) in different dimensions. F are spatial vectors that can cause substrate deformation both perpendicular to and within the viewing plane. (**a**) 2D TFM embedded with fluorescent microbeads; (**b**) substrate surface equipped with micropillar arrays; (**c**) 3D TFM embedded with fluorescent microbeads.

In traditional two dimensional (2D) TFM, markers near the surface of flat elastic substrates are used to indicate substrate deformations generated by cellular forces. For polyacrylamide gel substrates, fluorescent microbeads are commonly embedded near the gel surface to serve as markers (Figure 1a). For instance, Gardel et al. [45] reconstructed traction stresses by embedding high-density fluorescent microbeads into polyacrylamide hydrogel (PAA) substrates. They found a robust dual-phase correlation between traction force and F-actin speed. As another widely used material in TFM, polydimethylsiloxane (PDMS) can easily be micropatterned. By methods such as microcontact printing, the approach based on PDMS substrates allows surface micropatterns to serve as markers [46,47]. For example, Balaban et al. [47] developed a novel approach that combines micropatterning of PDMS substrates and GFP-tagged focal adhesions in live cells. By measuring the deformation of micropatterns, this method allows real-time and direct measurement of forces exerted by cells at individual adhesion sites. After a certain period of cell culture and migration, the deformation of substrates or distribution of microbeads in a stressed state can be observed by optical microscopy. In a typical experiment, a load-free image of microbead distribution is usually acquired via cell removal (e.g., trypsin) or cell lysis (e.g., RIPA) [48]. To avoid cell damage during experiments, Bergert et al. [49] developed a reference-free method based on nanodrip-printing of quantum dots as markers. By using computer algorithms, images of marker positions in the stressed and unstressed states can be analyzed precisely to determine marker displacement induced by cellular forces [48].

TFM based on flat elastic substrates allows unrestricted cell adhesion in space. With the optimization of technology and the development of high-resolution imaging techniques, the sensitivity of TFM has been greatly enhanced. At present, TFM has been widely used to measure cellular forces in various biological processes [50,51,52]. For instance, Barbieri et al. [53] significantly improved the resolution of planar cellular force probing by combining TFM with rapid two-dimensional total internal reflection fluorescence (TIRF) microscopy and structured illumination microscopy (SIM). This 2D TIRF-SIM-TFM methodology provides nano- and subsecond spatiotemporal resolution relevant to forces. This approach revealed the role of mechanical forces in various biological processes, including small and transient shearing forces during cell–ECM adherence of cervical cancer cells, the early stages of rat basophilic leukemia (RBL) cell activation, and the migration of primary salmonid keratocytes.

### 2.2. Two Dimensional Traction Force Microscope Based on Micropillar Arrays

Another simple alternative strategy is based on microfabricated arrays of flexible elastomer (such as PDMS) pillars (Figure 1b) [54,55]. The flexibility of pillars can be adjusted by controlling their geometric shape, such as the aspect ratio and width. Elastic micropillar arrays allow for decoupling substrate stiffness from adhesiveness and surface mechanical properties, enabling independent regulation [56]. When the pillars have a sufficiently high aspect ratio, they can be deformed under the action of cellular traction forces. Each pillar acts as an independent local force sensor. By tracking the bending of individual pillars, the local mechanical forces exerted by cells on the micropillar substrates can be quantified spatially and temporally [57]. The functionalization of the micropillar surface can typically be achieved with many methods such as microcontact printing, incubation, and surface nanopatterning [58,59]. The tops of the pillars can be chemically modified with cell adhesion ligands (such as fibronectin) or bioactive compounds for measuring cellular forces in various biological processes. For instance, Bashour et al. [60] measured the mechanical forces during T-cell activation by using costimulatory micropillar arrays coated with the T-cell receptor (TCR) coreceptors CD3 and CD28. Using microfabricated arrays conjugated with fibronectin by microcontact printing, Doss et al. [61] studied the organization of the cytoskeleton, traction forces, and stiffness response during the cell adhesion. In contrast to flat or continuous substrates, micropillar arrays provide topographical cues to cells and impose spatial restrictions on cell adhesion and growth.

### 2.3. Multidimensional Traction Force Microscope

However, cell traction forces are spatial vectors that can cause substrate deformation both perpendicular to and within the viewing plane. Deformations within the plane can be observed and measured by using traditional wide-field microscopy. Most of the existing measurement methods primarily focus on traction force within the viewing plane (2D) [34,62,63]. The traction forces of cell–matrix interactions are complex spatial vectors. Thus, measuring the component forces within the viewing plane is insufficient to accurately characterize the cellular traction forces. Advancements in high-resolution microscopes capable of 3D reconstruction such as confocal microscopes, have made it possible to track substrate deformations perpendicular to the viewing plane and promote the development of 2.5D and 3D TFM(Figure 1c). 2.5D TFM can be utilized to fully characterize 3D traction force fields on 2D substrates [64]. The key distinction between 2.5D TFM and 2D TFM lies in the methods used to acquire microscopy images. 2.5D TFM offers 3D positions of markers by using high-resolution microscopes such as confocal microscopes [40]. However, cells in vivo exist within 3D microenvironment, and research has shown significant differences between cell phenotypes in 3D microenvironment and those in 2D [65]. To fully characterize cell traction forces in their native environment, researchers proposed 3D TFM (Figure 1c), in which cells are embedded in substrates mixed with fluorescent microbeads [66]. The matrix materials used in 3D TFM include natural matrices (such as recombinant type I collagen [67,68]) or degradable polyethylene glycol synthetic hydrogels [66]. The Young’s modulus of the 3D substrate is typically in the range of 600–1000 Pa [66], which is similar to that of common extracellular matrices, such as recombinant collagen or Matrigel, as well as in vivo tissues such as breast and brain tissue [69,70]. In other experiments, materials with even lower Young’s moduli (200–300 Pa) have also been used [67,68]. 3D TFM is an important tool for exploring cellular mechanical behaviors in 3D microenvironment.

By tuning the elasticity of substrate materials, TFM can map the force distribution at cell–ECM surfaces with a wide dynamic measurement range and allow traction forces to be measured at different levels [71]. However, the intrinsic elasticity of TFM substrate materials determines the sensitivity of TFM (~nN) [72,73], while the density of standard markers or the pillar density and size of micropillar arrays determine the spatial resolution of the method (typically ~0.5 μm to several micrometers) [49,74,75]. Although TFM has been used to measure cellular forces in various applications, it is limited by the spatial resolution when measuring the forces generated by small cellular structures such as podosomes. Therefore, high-resolution techniques have been developed.

AFM has been proven to be a powerful tool for investigating cell structures at nanometer-scale resolution [76]. In the early stages, Labernadie et al. [77] combined AFM with correlative fluorescence microscopy and patterned substrates to characterize the biophysical properties of podosomes, such as height, hardness, and rheological properties. However, AFM cannot probe the basal tip of the podosome in contact with the substrate and is therefore unable to measure the protrusive forces of podosomes. By analogy with TFM, protrusion force microscopy (PFM) can be used to estimate podosome protrusive forces [78]. The indentation induced by the cell podosomes onto specially fabricated, compliant Formvar substrates was measured with AFM, and the protrusive forces were then calculated using a model based on the mechanical properties of the substrates. Maridonneau-Parini research group has performed a series of studies related to podosomes using PFM. For instance, by using time-lapse PFM, Proag et al. [79] found that the protrusion force of the first neighbor of the foot body changes in a synchronous manner, indicating that there is a short-range interaction that regulates their mechanical activity. Bouissou et al. [80] demonstrated that the podosome ring balances protrusion at the core as a tension site. By opposing forces, the podosome can serve as a nanoscale autonomous force generator. Due to the challenges in applying PFM to whole-cell measurements of tangential traction forces, PFM is a highly specialized technique for measuring protrusive forces and serves as a complementary method to TFM.

In addition, two other methods for converting forces into strain with high precision are elastic resonator interference stress microscopy (ERISM) and photonic crystal cellular force microscopy (PCCFM). By measuring the microcavity deformation generated by cell traction forces, ERISM can map stress with 2 nm displacement resolution and 1 pN force sensitivity, enabling the investigation of forces exerted by small structures such as individual invadopodia [81,82,83]. ERISM offers a robust approach to measure vertical mechanical forces at cell-substrate surfaces. The optical microcavity substrate consists of two semitransparent gold mirrors with an ultra-soft elastomer sandwiched between them, and the top mirror is coated with ECM proteins. The mechanical forces exerted by cells cause local vertical deformations of the microcavity, leading to local shifts in the resonance wavelength. Subsequently, based on the local deformations of the microcavity, high-resolution stress maps can be obtained via optical models and finite element analysis. ERISM eliminates the need for a zero-stress reference image, allowing time-lapse imaging and immunostaining with cells on substrates [81]. However, due to the requirement of a stress-free region as a boundary condition, ERISM cannot measure cellular forces at the tissue level (such as in monolayers), which hinders the study of cellular mechanical behavior across multiple scales [84]. Additionally, the significant differences between the gold-coated elastic substrate and in vivo biopolymers may interfere with the mechanical interactions between cells and substrate [85,86]. However, with extremely high precision over long periods, ERISM can serve as a complementary technique to TFM, particularly for measuring changes in substrate thickness induced by cells. Unlike ERISM, PCCFM can measure tiny vertical cell forces without the need for reference or boundary conditions. By using artificial photonic crystal hydrogel substrates, micro/nano-deformation induced by cells can be converted into perceivable color changes [87,88], allowing the imaging and quantification of vertical cellular forces [89]. Li et al. [89] developed a PCCFM system to rapidly detect mechanosensitive subcellular structures, such as focal adhesions (FAs). This method achieves high-throughput measurements and provides vertical force information at the subcellular, cellular, and tissue levels within a single image. The nanoparticles or hydrogels used for substrates are not only easy to obtain and cost-effective but also allow for the customization of specific substrates according to the usage environment. However, although PCCFM enables measurement of vertical cellular forces, it is currently not applicable for three-dimensional measurements. Moreover, PCCFM enables the measurement of vertical cellular forces and does not directly substitute for TFM in providing comprehensive mechanical insights.

## 3. Fluorescent Molecular Force Sensors

Many force-transmitting structures in cells, such as podosomes and focal complexes, are mostly on the micron or submicron scale, with forces ranging from piconewton to nanonewton [90]. Although TFM can be used to directly map cellular traction forces, its limitations in spatial resolution and force sensitivity restrict its capacity to reconstruct the molecular mechanical forces with comparable precision [74,91]. Over the past decade, a series of FMFS has been developed for measuring cell traction forces generated by specific molecules within cells at the nanoscale. These FMFS convert cellular mechanical phenotype into fluorescent signals, thus enabling high-resolution visualization and quantification of cellular forces in various mechanobiological processes. FMFS generally consists of four components: (1) ligands that can bind to cell membrane receptors or cholesterol molecules; (2) force-sensitive elements; (3) fluorescent reporter systems, including typical Förster fluorescence resonance energy transfer (FRET) fluorophore pairs, fluorophore–quencher pairs, or fluorophores; and (4) a substrate-binding site for anchoring the probe. When cellular forces act upon FMFS, structural changes (extension, rupture, or unfolding) in force-sensitive elements occur within the FMFS, thus altering the state of the fluorescent reporter system. The magnitude of cell traction forces is reported by changes in FRET efficiency, fluorescence enhancement, fluorescence emission characteristics, or fluorescence loss. Based on these principles, FMFS can achieve the conversion of force signals into fluorescence signals, which enhances spatial resolution and measurement sensitivity in force imaging.

### 3.1. The Principle of Force-to-Fluorescence Conversion

#### 3.1.1. Förster Fluorescence Resonance Energy Transfer

**Figure 2:**
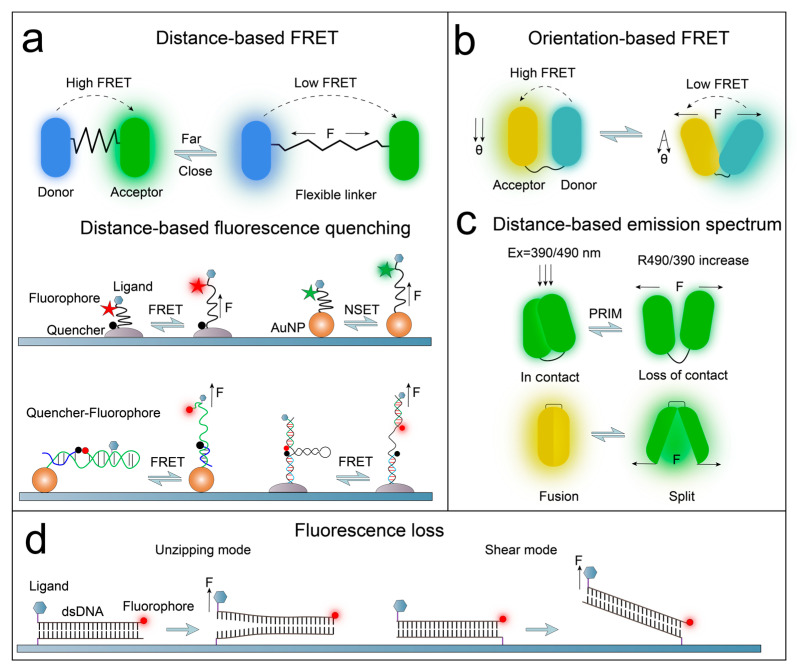
Overview of force-to-fluorescence conversion based on different principles: (**a**) distance-based FRET or fluorescence quenching, adapted from Refs. [102,103,104,105]; (**b**) orientation-based FRET, adapted from Ref. [95]; (**c**) distance-based emission spectrum, adapted from Refs. [106,107,108]; (**d**) fluorescence loss, adapted from Ref. [109].

With the ability to measure distances at the nanoscale, FRET can be used as a “molecular ruler” to detect biomolecular structures [92], and it provides real-time in situ detection in mechanical sensors. FRET refers to the nonradiative energy transfer that occurs through dipole–dipole interactions between an excited state donor (D) fluorophore and a ground state acceptor (A, another fluorophore or quencher) when there is appropriate spectral overlap or proximity (Figure 2a) [93]. Within the effective range, the fluorescence signal of typical FRET pairs is highly sensitive to conformation, binding, and dissociation of molecules [94]. The conditions for effective energy transfer between D and A are stringent. FRET efficiency E is influenced by several factors such as the degree of overlap between donor emission and acceptor absorption spectra J(λ), the separation distance r between the D–A pair (typically within the 1−10 nm range), and the relative orientation of the D–A transition dipoles [95] (i.e., the angle between the donor and acceptor) [96]. According to the model used to describe the distance of a single D–A pair [97], E is highly dependent on r:

(1) $$E=\frac{R_{0}^{6}}{R_{0}^{6}+r^{6}}$$

In Equation (1), $R_{0}$ is the Förster distance for the given D–A pair, which is r when E equals 50%. $R_{0}$ depends on the relative orientation of D and A transition dipoles $\kappa^{2}$, the refractive index n of the medium between the D–A pair, the quantum yield $Q_{D}$ of D, and the spectral overlap integral J(λ):

(2) R0=0.0211(κ2n−4QDJ(λ))16

From the above formula, it is evident that E is highly sensitive to minute changes in r, and it may be significantly influenced by the relative orientation of the D–A dipoles. Currently, methods used to determine E include measurements based on fluorescence intensity, fluorescence lifetime, or anisotropy [98]. Therefore, based on a D–A pair, FMFS can convert cell traction forces into measurable changes in E.

- Distance-Based FRET Fluorophore Pair

Elastic molecules, such as single-stranded DNA, can serve as pN force sensors when inserted between D−A pairs [99]. When the elastic molecule elongates with forces, the distance between the D–A pair increases, leading to a decrease in E (Figure 2a). Genetically-encoded fluorescent proteins are commonly used as D–A pairs in intracellular protein mechanical force sensors because of their advantages such as higher precision in biomolecular labeling and minimal perturbation to biomolecular structure/function [93]. For example, a fluorescence resonance energy transfer cassette (stFRET) designed by Meng et al. [100], can be inserted into cytoskeletal protein hosts (such as α-actinin, non-erythrocyte spectrin, and filamin A) for measuring the in situ mechanical stress of structural proteins within cells. Compared with genetically-encoded fluorescent proteins, organic dyes are more photostable and can be combined with multifunctional surface chemistry techniques. Thus, they are commonly used to measure mechanical force on the cell surface. For instance, Chang et al. [101] constructed a molecular tension sensor (MTS) with spider silk peptide as the force-sensitive element and the dyes Alexa546 (donor) and Alexa647 (acceptor) as the D–A pair. The change in the FRET efficiency of Alexa546–Alexa647 reports the traction forces of individual integrins in living cells. This fluorescent reporter system indicates that a low FRET efficiency represents high mechanical forces.

- Distance-Based Fluorescence Quenching

Due to the wide absorption spectrum of quenchers and the lack of fluorescence emission, fluorophore−quenchers exhibit more obvious binary “on/off” energy transfer behavior (Figure 2a). For example, Stabley et al. [102] inserted polyethylene glycol (PEG) between a specific fluorophore (Alexa Fluor 647) and a quencher (QSY21), designing a force sensor to measure the force transmission of EGFP. Cellular mechanical forces extend the elastic molecule PEG (surface-modified with quencher), removing the fluorophore away from the quencher, thereby leading to an increase in fluorescence intensity and reporting the mechanical forces applied by the cell. This sensor serves as a simple “on” signal readout without the need to perform corrections for spectral bleed-through or cross talk. Compared with traditional molecular quenchers, gold nanoparticles (AuNPs) exhibit superior fluorescence quenching abilities. Due to nanometal surface energy transfer (NSET), AuNPs can be used for highly sensitive detection of molecular distances [110,111]. Theoretical and experimental studies have shown that the surface energy transfer behavior (i.e., quenching efficiency) of AuNPs (with diameters in the range of 1–20 nm) is inversely proportional to the fourth power of the distance between the fluorophore and AuNPs. The effective quenching distance of NSET can reach tens of nanometer, indicating that AuNPs are suitable as nanoscale quenchers for FMFS [112,113]. For instance, Liu et al. [103] replaced the synthetic quencher of molecular tension fluorescence microscopy (MTFM) with AuNPs, broadening the dynamic measuring range of the mechanical sensor and enhancing its signal-to-noise ratio and force sensitivity.

- Orientation-Based FRET

From Equations (1) and (2), it is clear that FRET efficiency relies on the relative orientation of D–A pair transition dipoles, allowing for the development of orientation-dependent FRET force sensors. Meng et al. [95] designed an orientation-based genetically encoded protein force sensor named cpstFRET (Figure 2b). They used a peptide to link two circularly permuted fluorescent proteins, cpCerulean and cpVenus [114]. cpstFRET can detect cellular mechanical force via changes in FRET efficiency mediated by the relative angle between cpCerulean and cpVenus. cpstFRET has a wide dynamic range and is physically small (54 kDa), minimizing its impact on the host protein. However, the design of orientation-based FRET sensors requires extensive effort due to their complexity.

#### 3.1.2. Properties of Fluorophore Emission 

In contrast to FRET, physical contact between two GFP modules may cause structural perturbations within the modules and accompanying emission spectral changes. The method based on this principle for imaging is known as proximity imaging (PRIM) [115]. Based on PRIM, the strain-sensing module (PriSSM) designed by Iwai et al. [106,107] consists of a tandem fusion of normal GFP and circularly permuted GFP (Figure 2c). When external forces result in the loss of partial contact between GFP modules, PriSSM will display fluorescence characteristics similar to those of monomeric GFP, with stronger green fluorescence intensity produced upon excitation at 490 nm. PriSSM reports mechanical forces by changes in the ratio of emission intensities produced upon excitation at 390 nm and 490 nm. Yellow fluorescent protein (YFP) is a variant of GFP with a chromophore composed of GFP and the phenolic group Tyr203 [116], and its emission spectrum depends on the stacking distance between the chromophore and phenolic group [117]. Ichimura et al. [108] constructed a circularly permuted YFP (cpYFP) and fused a β-hairpin peptide as a linker to the N and C termini of cpYFP. When a force is applied between the N and C termini, the distance between the chromophore and Tyr203 changes, leading to alterations in the fluorescence spectrum. The structure-mediated fluorescence emission characteristics of cpYFP reflect the mechanical force experienced by the sensor (Figure 2c).

#### 3.1.3. Loss of Fluorescence

As one of the most commonly used molecular force sensors, fixed force sensors are immobilized on solid supports to measure the forces between cell membrane receptor–ligand or cell–cell interactions. If force sensors tagged with fluorophores undergo deformation or disruption under cellular forces, the fluorescence loss on the surface of the solid support can reflect cellular mechanical phenotypes. Wang et al. [109] used streptavidin–biotin bonds to immobilize the bottom strand of tension gauge tethers (TGT) onto the substrate surface, with Cy3 fluorophores and integrin ligands conjugated to the top strand of the double-stranded DNA. When cellular forces exceed the tension tolerance (T_tol_) of TGT, the rupture of TGT leads to fluorescence loss on the substrate surface, indicating the magnitude and distribution of cellular forces (Figure 2d).

### 3.2. FMFS Based on Different Force-Sensitive Elements

The mechanical properties of force-sensitive elements determine the fluorescence readout, which is crucial for achieving high-resolution imaging of cell traction forces. Force-sensitive elements typically possess unique force–extension mechanical properties and need to be matched with the effective distance required by the fluorescent reporter system. The linker serving as a force-sensitive element can be an entropic spring (such as PEG), contain some degree of structure (such as peptides, proteins), or a nucleic acid molecule with a defined structure (such as double-stranded DNA, DNA hairpin).

In 1999, through AFM experiments, Oesterhelt et al. [118] found that PEG polymers possess remarkable and reversible force–extension curves. With excellent mechanical properties [118,119], chemical accessibility in molecular engineering, outstanding biocompatibility [120], and stability [121], PEG has become a widely used force-sensitive molecular spring. For instance, Stabley et al. [102] used PEG as a force-sensitive element to develop a MTFM. Single-molecule force spectroscopy has revealed that spider silk peptide can be used as a molecular entropy nanospring suitable for force measurements at the piconewton level [90]. Using Spider silk peptide as a force-sensitive element, FRET-based FMFS can detect single-molecule forces within 1–6 pN [122,123]. Alexander R. Dunn research team constructed a molecular tension sensor (MTS) with spider silk peptide as the force-sensitive element, utilizing changes in FRET values to report low-level dynamic traction forces (1–6 pN) [101,124,125]. Experimental and theoretical modeling studies have shown that the 27th immunoglobulin domain of cardiac titin (I27) has highly reversible force–extension curves and higher unfolding forces [126]. Galior et al. [127] used I27 to link fluorophores with AuNPs, constructing FMFS capable of detecting higher levels of forces (beyond ~30 to 40 pN). The addition of disulfide bonds to I27 prevented mechanical unfolding, and significantly expanded the detection range of FMFS.

Compared to other polymers, DNA enjoys established chemical synthesis and modification methods. With the programmability of force responsivity, DNA is one of the preferred choices for force-sensitive elements [128,129]. The base sequence and the position of the force load determine the tension tolerance of DNA [130,131]. The dynamic measuring range of dsDNA-based TGT is approximately 12–56 pN [132]. Compared to dsDNA, DNA hairpin (single-stranded DNA, ssDNA) exhibits superior folding efficiency and dynamic properties, with force-response behavior more similar to a reversible digital switch. Li et al. [104] developed a reversible shearing DNA-based tension probe (RSDTP). The tension tolerance of RSDTP can be adjusted by modifying the GC content and force configuration within the DNA hairpin (4–60 pN). Furthermore, the reversible state of RSDTP can be transformed into an irreversible state through photolytic induction, transitioning from a DNA hairpin sensor to a TGT sensor.

### 3.3. Applications of FMFS in Various Biological Processes

#### 3.3.1. Intracellular Mechanical Force Transmission of Protein Molecules

**Figure 3:**
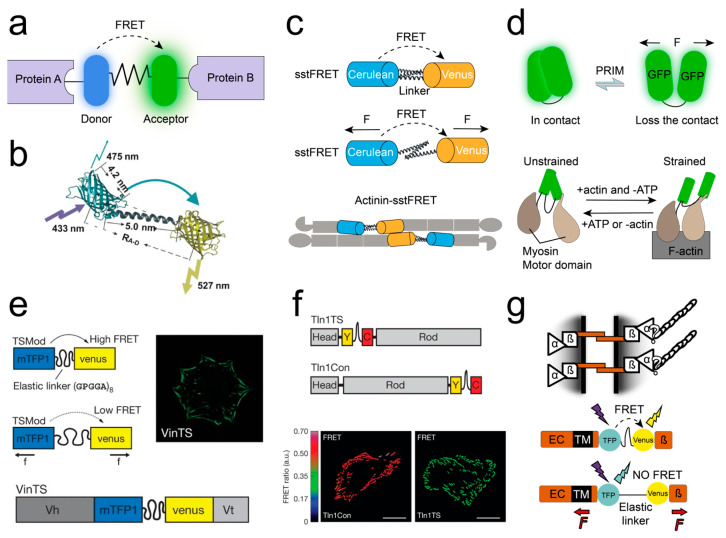
Examples of FMFS for measuring intracellular mechanical forces. (**a**) General schematic of genetically encoded FMFS. (**b**) FRET cassette (stFRET), which can be inserted into host structural proteins within cells to observe in situ strain [100]. Copyright 2008, reproduced with permission from John Wiley and Sons. (**c**) Spectrin stFRET (sstFRET), utilized for reporting mechanical forces on the cellular α-actinin protein, adapted from Ref. [134]. (**d**) PriSSM, designed for myosin–actin interaction, adapted from Refs. [106,107]. (**e**) TSMod for measuring mechanical forces on vinculin [122]. Copyright 2010, reproduced with permission from Springer Nature. (**f**) FMFS, designed for measuring mechanical forces on talin. Scale bars: 20 μm [136]. Copyright 2015, reproduced with permission from Springer Nature. (**g**) TSMod-based E-cadherin tension sensor (EcadTSMod), adapted from Ref. [137].

FMFS used for measuring the mechanical force transmission of intracellular proteins typically belong to genetically-encoded force sensors, where the fluorescent reporter system is based on FRET fluorescent protein pairs. These force sensors can be inserted into target proteins via genetic engineering techniques, allowing for the measurement of mechanical stress experienced by the target protein without affecting its normal function (Figure 3a).

- Cytoskeletal Stress

As previously mentioned, stFRET developed by Meng et al. [100] can be inserted into several types of intracellular structural protein hosts (such as α-actinin, non-erythrocytic spectrin, and filamin A) and expressed in cultured cells to observe in situ stress of structural proteins within living cells (Figure 3b). However, because calibration of stFRET was not included in previous work, the forces observed could not be quantified. To enhance the sensitivity of stFRET and ensure its mechanical compliance similar to that of host proteins, Meng et al. [133] replaced the α-helical peptides in the original system with spectrin repeat sequences and named it spectrin stFRET (sstFRET). sstFRET exhibits sensitivity to forces in the range of 5–7 pN. It can be inserted into α-actinin and expressed in cells to serve as a tool for measuring internal stresses within the cell. Furthermore, Susan Z. Hua research team used sstFRET to observe cytoskeletal stresses induced by shear flow (Figure 3c) [134] and measured the time dependence between α-actinin tension and FAs dynamics [135].

- Cellular Signaling Pathways

Iwai et al. [106,107] designed PriSSM for visualizing the mechanical interactions between myosin and actin within cells (Figure 3d). Grashoff et al. [122] used the spider peptide (GPGGA)_8_ to link the fluorescent protein pair mTFP1–Venus as the tension sensor module (TSMod, 1–6 pN) to assess the intracellular traction forces generated by the structural protein vinculin during cell adhesion and migration processes (Figure 3e). They found that vinculin is indispensable in stabilized FAs, with an average tension of approximately 2.5 pN. However, in disassembling or sliding FAs at the rear of migrating cells, tension is low. Subsequent research demonstrated that improving the fusion compatibility of FMFS based on (GPGGA)_8_ with intracellular target proteins could enable the analysis of mechanical forces during interactions among different proteins within living cells [123]. To measure higher levels of cellular traction force, Austen et al. [136] replaced the spider silk peptide in TSMod with HP35 peptide and its mutants (HP35st) as force-sensitive elements (Figure 3f). This modification extended the effective measurement range of FMFS to 6–8 pN and 9–11 pN, enabling the measurement of mechanical forces generated by talin. These two kinds of FMFS revealed that the integrin activator talin establishes mechanical linkages during cell adhesion and bears an average mechanical force of 7–10 pN. Isoform talin regulates the sensitivity of cells to extracellular rigidity. To explore whether the mechanical force between cells is directly transmitted through cadherins, Borghi et al. [137] developed an E-cadherin tension sensor based on TSMod (EcadTSMod). EcadTSMod can be used to measure the forces experienced by the cytoplasmic domain of E-cadherin at cell–cell contacts and in noncontact areas of the plasma membrane (Figure 3g).

#### 3.3.2. Cell–ECM Interactions

FMFS applied to measure the forces between specific receptors on the cell membrane and matrix ligands are typically fixed force sensors that need to anchor to ECM surfaces for detection.

- Cell Endocytosis

**Figure 4:**
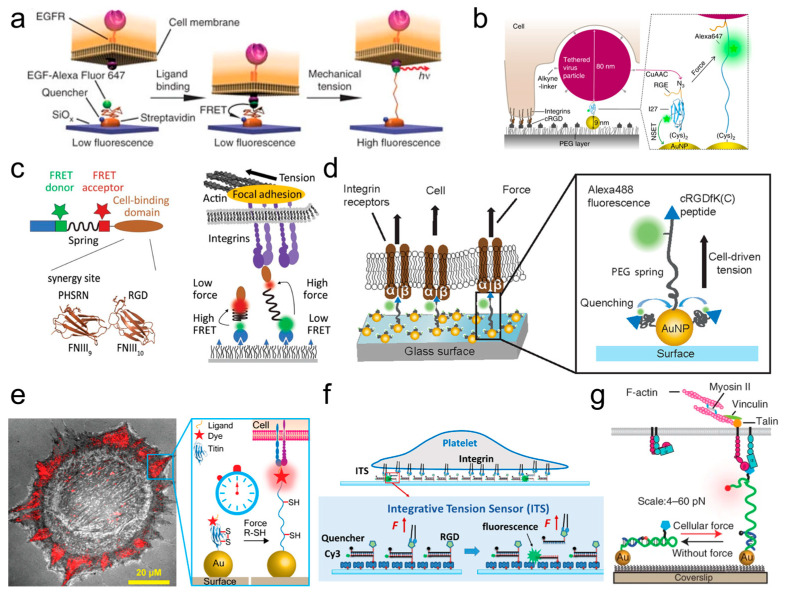
Examples of FMFS for measuring cell–ligand mechanical forces. (**a**) FMFS for measuring the mechanical force on EGFR during the early stages of endocytosis [102]. Copyright 2011, reproduced with permission from Springer Nature. (**b**) FMFS for measuring the mechanical force during the process of host cell uptake of viral particles [138]. Copyright 2020, reproduced with permission from Springer Nature. (**c**–**g**) FMFS used for measuring the mechanical forces during the cell adhesion process: (**c**) FMFS based on peptides [101], copyright 2016, reproduced with permission from American Chemical Society; (**d**) FMFS based on PEG [103], copyright 2013, reproduced with permission from American Chemical Society; (**e**) FMFS based on I27. The mechanical forces transmitted by integrins extended the I27 protein, leading to an increase in the intensity of the dye (stars). [127], copyright 2016, reproduced with permission from American Chemical Society; (**f**) FMFS based on dsDNA [142], copyright 2017, reproduced with permission from Elsevier Publisher; (**g**) FMFS based on ssDNA [104], copyright 2021, reproduced with permission from Springer Nature.

Stabley et al. [102] reported the first fixed-FMFS targeting cell surface receptors in time and space. This FMFS can precisely assess the mechanical forces of epidermal growth factor receptor (EGFR) activated by epidermal growth factor (EGF) during the early stages of endocytosis (Figure 4a). Through ligands (coupled with Alexa Fluor 647), cell traction forces extended the flexible linker PEG and separate the fluorophore from the quencher. FMFS reported the dynamic mechanical forces involved in the interaction between EGFR and EGF. Wiegand et al. [138] designed FMFS to measure the dynamics of cell forces during the uptake of virus particles by host cells. They anchored one end of the I27 protein with AuNPs on the substrate surface, with the other end coupled to a fluorophore and virus particles (Figure 4b). This method revealed that the average traction force exerted by adherent cells exceeded 30 pN during the process of viral uptake from the ventral side. Zhang et al. [139] described a platform for exploring the mechanical forces during viral infection. This platform anchors the virus on a multivalent controlled aptamer, allowing the transmission of mechanical force between the virus–cell complex to TGT. This method offers a new perspective for detecting the mechanical forces of different viral variants.

- Cell Adhesion and Migration

As previously mentioned, Alexander R Dunn research team constructed MTS based on spider silk peptide. MTS reported low-level dynamic traction forces (1–6 pN) exerted by different integrins on specific ligands through changes in FRET values (Figure 4c) [101,124,125]. Liu et al. [103] developed a MTFM based on PEG. MTFM measured cell traction forces at the piconewton level, imaged cell integrin adhesion dynamics, and revealed that the cell integrin–ligand traction forces ranged from 1 to 15 pN, with an average traction force within FAs of approximately 1 pN (Figure 4d). To measure the upper limit of cellular integrin traction forces, Galior et al. [127] constructed a MTFM based on I27 capable of detecting 30 to 40 pN integrin–ligand forces. The addition of disulfide bonds significantly increased upper detection limit (~80 to 200 pN) of MTFM. When cell traction forces extend MTFM to expose the hidden disulfide bond, the reducing agent dithiothreitol (DTT) in the medium will open the disulfide bond, leading to the extension of the protein chain and dequenching of the fluorophore. The nanoparticle titin force sensor revealed partial integrin tensions of 110 ± 9 pN at high levels within the FAs of rat embryonic fibroblasts (Figure 4e).

Wang et al. [132] first used TGT to measure single integrin–ligand traction force during cell adhesion, as well as the traction forces required for Notch receptor activation. When the traction force applied by cells through integrins exceeds the tension tolerance (T_tol_) of TGT, TGT ruptures and does not activate the downstream receptor signaling pathway. Otherwise, the signaling pathway is activated. This experiment indicated that at the early stage of adhesion formation between cells and TGT (with different T_tol_), single integrin exerted a force of approximately 40 pN on the ligand, while the mechanical force required for Notch receptor activation is less than 12 pN. Moreover, TGT labeled with fluorophores (e.g., multiplex TGT ) or TGT labeled with fluorophore–quencher pairs (such as ITS or qTGT) can be used for imaging and measuring the traction force of cell integrins, revealing different levels of integrin–ligand forces during cell adhesion [109,140] and migration [141]. Among these, multiplex TGT (mTGT) [109], which has a wide detection range (10~60 pN) can simultaneously monitor different levels of integrin traction forces. mTGT revealed that the integrins outside FAs also transmitted traction forces after FA formation, and these forces were mainly distributed in the range of 43−54 pN, lower than the integrin forces inside FAs. Wang et al. [142] used integrative tension sensor (ITS) to map the distribution of integrin traction forces generated by platelets, discovering the distribution of polarized forces during the platelet adhesion (Figure 4f). Zhao et al. [141] used ITS to measure integrin tensions during keratinocytes migration. They found that migrating keratinocytes applied 50–100 pN tensions at the cell rear. These tensions can disrupt local integrin–ligand bonds and disassemble FAs, thereby mediating cell detachment from substrates.

DNA hairpin labeled with fluorophore–quencher pairs can serve as a reversible force-responsive switch. This switch can be used for real-time imaging of traction forces during cell adhesion. When forces applied by cells through ligands on the DNA hairpin exceeds its fracture threshold, the DNA hairpin is unzipped but maintaining strand integrity, and the fluorescence signal intensifies. Once that traction force is removed, the unfolded DNA hairpin can return to its original structure, and the fluorescence ceases. Based on DNA hairpins, Blakely et al. [143] developed a molecular tension probe (TP) with a force threshold range of 5.7–16.5 pN. The 3′ end of DNA hairpin (attached to the quencher) is anchored to the substrate surface, and the 5′ end is coupled to adhesive peptides via PEG. TP allows for reversible optical measurement of cell traction forces in both space and time, revealing spatial heterogeneity in the distribution of traction forces among and within different FAs during cell adhesion. Zhang et al. [105], utilizing DNA hairpin as “switch” elements, designed a three-stranded system MTFM digital tension probe. Real-time imaging with three-stranded system MTFM showed that integrin tension is highly dynamic and increases with the density of integrins during the formation of cell adhesion. The experiment indicated that integrins have a mechanical preference for cyclic RGD peptides over linear RGD peptides. To achieve reversible measurements of higher-level receptor–ligand mechanical forces (>20 pN), Li et al. [104] developed RSDTP with the range of 4–60 pN (Figure 4g). RSDTP revealed the crucial role of integrins during the cell adhesion process, where integrins in weak-force adhesion trigger FA formation, whereas load-bearing integrins in strong-force (>56 pN) adhesion maintained FA structure and promoting maturation.

To avoid deoxyribonuclease (DNase) damage to DNA-based FMFS, Zhao et al. [144] designed a FMFS based on peptide nucleic acid (PNA)/DNA hybrid which retains the ability to convert force into fluorescence and remains stable in cells expressing DNase. This DNase-resistant tension sensor can report cellular traction forces of various cell types in real time and stably, broadening the application scope of molecular mechanosensors for cellular mechanics. To solve the restriction of large-scale equipment and high experimental manipulation, Amouzadeh et al. [145] developed a novel DNA-based electrochemical force sensor. This sensor enables high-sensitivity, stable, simple, and portable measurements of cellular traction forces. TGT and DNA hairpin are anchored onto the surface of a gold wire mesh printed electrode. Cell adhesion force of specific receptor–ligand interactions can separate TGT or unfold the DNA hairpin, resulting in a reduction in electrochemical signal. And the change in electrochemical signal can be monitored and recorded in real time using a smartphone.

#### 3.3.3. Monitoring and Regulating Cellular Mechanical Functions

- Maturation of Cardiomyocytes

**Figure 5:**
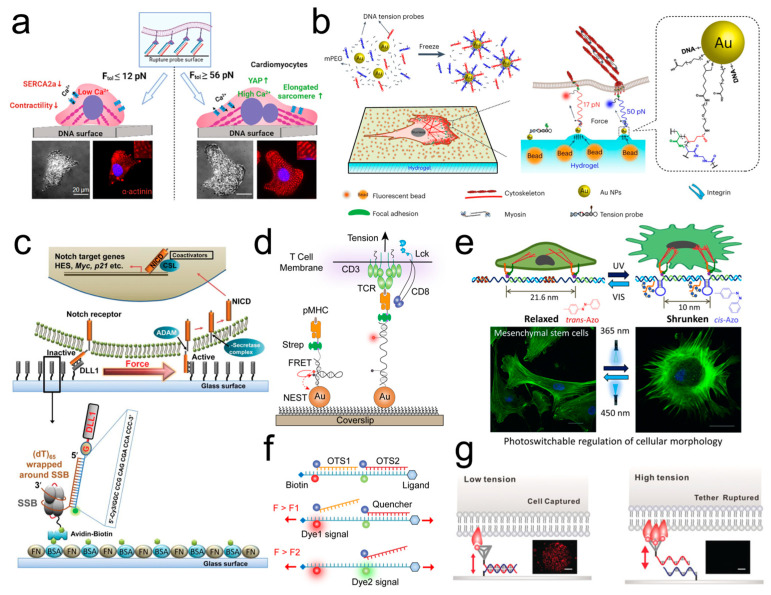
Examples of FMFS for monitoring and regulating cellular mechanical functions. (**a**) FMFS used for monitoring the maturation of cardiomyocytes [91]. Copyright 2022, reproduced with permission from American Chemical Society. (**b**) FMFS employed for monitoring receptor-mediated rigidity sensing [146]. Copyright 2023, reproduced with permission from Springer Nature. (**c**) FMFS designed for monitoring the activation of Notch [147]. Copyright 2016, reproduced with permission from American Chemical Society. (**d**) FMFS designed for monitoring the activation of T cells, adapted from Ref. [148]. (**e**) FMFS used for regulating cell morphology and motility [149]. Copyright 2021, reproduced with permission from Wiley-VCH GmbH. (**f**) FMFS developed for monitoring single-molecule loading rate during cell adhesion, adapted from Ref. [150]. (**g**) FMFS utilized for sorting cell mixtures [151]. Copyright 2020, reproduced with permission from Wiley-VCH GmbH.

Rashid et al. [91] utilized TGT with different tension tolerance T_tol_ (~12, ~56, and ~160 pN) to regulate and monitor cardiomyocyte maturation (Figure 5a). Their research indicated that adhesive TGT with higher T_tol_ can promote cardiomyocytes maturation, revealing the significant roles of pN integrin forces in the early stages of heart development and cell differentiation.

- Receptor-Mediated Rigidity Sensing

To further investigate the molecular mechanisms of cellular rigidity sensing and mechanotransduction, Wang et al. [146] developed a hydrogel-based FMFS that combines molecular tension fluorescence microscopy (mTFM) with TFM to explore the regulation of cellular receptor-mediated force transmission by stiffness (Figure 5b). They prepared stable DNA–AuNP comlexes by linking DNA-based tension probes with AuNPs and then immobilized these DNA–AuNP comlexes on the surface of polyacrylamide hydrogels coated with fluorescent nanobeads via chemical cross-linking. The experiment demonstrated that fibroblasts respond to substrate stiffness by promoting FAs maturation. Substrate stiffness promoted T-cell activation by increasing the mechanical force transmitted by T-cell receptor (TCR) and the mechanical sampling frequency of TCR.

- Activation of Notch

To measure low-level single-molecule force (below 12 pN) required for the activation of Notch (cell surface receptors), Chowdhury et al. [147] developed a novel low-tension gauge tether (LTGT) based on the low dissociation force (~4 pN) between ssDNA and *Escherichia coli* ssDNA-binding protein (SSB). DLL1 (Notch ligand)–LTGT coupled with Cy3 fluorophore, is anchored to the substrate surface. When the ssDNA in DLL1-LTGT unspools from SSB under cellular traction forces, a loss of fluorescence signal can be detected (Figure 5c). They discovered that the mechanical force required to activate Notch ranged from 4 to 12 pN (with a loading rate of 60 pN/s).

- Activation and Aggregation of Platelets

Zhang et al. [152] cultured platelets on substrates connected to TGT with different tension tolerance T_tol_ and discovered that ligand mechanics can regulate the activation of initial platelets. Concurrently, using a three-stranded system MTFM digital tension probe to map out the distribution of integrin tension during the platelet activation process, they found that integrin tension is related to the early stages of platelet activation and synchronizes with calcium ion flux. The piconewton mechanical forces produced by integrins regulate platelet aggregation.

- Activation of Immune Cells

Based on DNA hairpin, Liu et al. [148] designed a nanoparticle FMFS to directly image and quantify the mechanical tension transmitted by TCRs during T-cell activation (Figure 5d). They discovered that the generation of TCR mechanical force was dependent on the involvement of CD8 coreceptor and adhesion ligands. Spillane et al. [153] constructed a DNA-based FMFS for reporting intracellular and extracellular enzymatic antigen degradation in B cells. The experiment indicated that B-cell antigen extraction primarily relies on mechanical forces, and the efficiency of mechanical extraction depends on the substrate stiffness, antigen tether strength, and B-cell receptor (BCR) affinity. Ma a et al. [154] developed a new FMFS capable of integrating and storing dynamic mechanical information. They utilized this FMFS to image transient and infrequent mechanical events during the antigen recognition process by TCRs. A locking strand (oligonucleotide) which can selectively hybridize with the mechanically unfolded hairpin was introduced into the structure of three-stranded system MTFM to prevent DNA refolding, mediating the storage of mechanical events. The thoughtfully designed unlocking strands allow the FMFS to arbitrarily switch between locked and unlocked states to record and erase molecular force signals, thereby enabling the selection of different time windows for force signal integration. To specifically activate T cells proliferation in tumors, Zhang et al. [155] developed a pH-driven interlocking DNA nano-spring (iDNS). iDNS can be used to regulate the nanoscale distribution of CD3 receptors through pH-driven reversible restructuring. The tumor environment with low pH drives the spring-like contraction of iDNS, promoting T-cell proliferation and enhancing antitumor effects. This method provides a therapeutic tool to reduce autoimmune side effects in antitumor immunotherapy.

- Regulation of Cell Motility and Morphology

Cell membrane receptors work in complexes during the process of sensing external factors. The activation of these receptors highly depend on nanoscale arrangement and the aggregation or disaggregation. Karna et al. [156] utilized DNA origami nano-springs to regulate cell motility by targeting the aggregation of integrins. The formation (at mildly acidic pH) and disassociation (at neutral pH) of i-motif structures within the DNA springs are regulated by pH, thereby achieving coiling or uncoiling of the nano-springs. When the nano-springs coil, the movement of HeLa cells was inhibited. When the nano-springs uncoil, the mechanical movement of cells is restored. Through AFM, Sethi et al. [149] discovered that DNA polymers can reversibly switch between the two different structures: a relaxed linear structure and a contracted dense structure. On this basis, they designed a light-responsive DNA polymer with an azobenzene switch (Figure 5e). This polymer can reversibly switch between relaxed and dense structures dependent on the wavelength of light excitation, dynamically and reversibly changing the distribution of RGD, thereby regulating cellular morphology. Additionally, Zhang et al. [157] developed a multivalent ligand material based on DNA nano-springs. The DNA nano-spring structure can reversibly change the spacing of RGD sequences through the addition of external DNA sequences (strand displacement reactions), thereby triggering the aggregation and disaggregation of integrin, and thus regulating intracellular signaling pathway and cell morphology.

- Determination of the Cell Traction Force Loading Rate

To resolve the dependence of FMFS on the force-loading rate in single-molecule force spectroscopy technologies, Zhao et al. [150] developed a DNA-based overstretching tension sensor (OTS) that can be used to accurately measure force in physiological conditions (Figure 5f). In the structure of OTSs, the 5’ end of ssDNA1 is coupled with a ligand that can bind to integrins. The 3’ end of ssDNA1 is connected to a Cy3 fluorophore and anchored to the substrate surface, while the 5’ end of the complementary strand ssDNA2 is coupled with a fluorescence quencher. The fluorescence signal is suppressed until the cellular traction forces remove the complementary quencher strand, leading to dehybridization. To measure force-loading rate, Atto647N-labeled OTS1 and Cy3-labeled OTS2 were serially connected. Using the decay time of the fluorescence signal generated by the dehybridization of OTS under tension and the increase in tension per single integrin, the single-molecule force-loading rate via integrins can be determined. The experiment revealed that the mechanical loading rate of cellular integrin ranged from 0.5 to 4 pN per second.

- Sorters of Cell Mixtures

To investigate the influence of topological structures on ligand–receptor binding strength, Yin et al. [151] anchored topological ligands based on tetrahedral DNA frameworks (TDF) to the substrate surface via TGT to examine the binding strength of different topological ligands (Figure 5g). When the mechanical forces transmitted by ligand–receptor interactions exceed T_tol_, TGT rupture resulted in the inability of cells to adhere to the substrate, thereby being washed away and removed. By tuning the ligand–receptor binding strength, cell sorting in cell mixtures can be achieved.

#### 3.3.4. High-Throughput Cellular Mechanobiology Analysis Platform

- Combination with ELISA Reader

**Figure 6:**
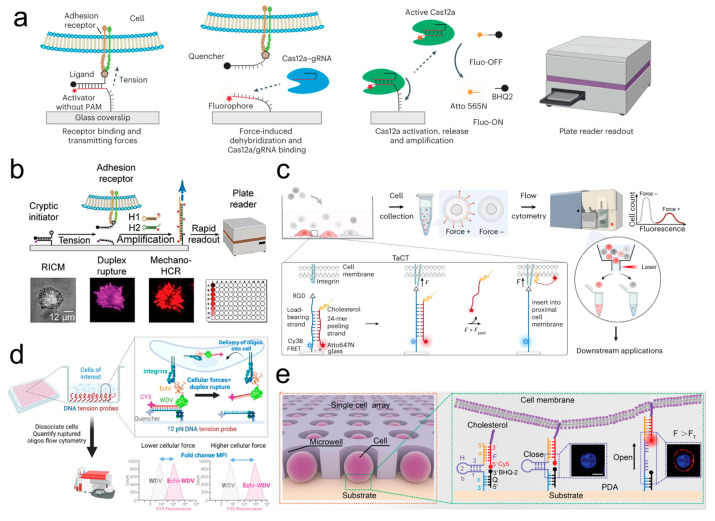
Examples of FMFS applications in high-throughput screening platforms. (**a**) Schematic showing the mechano-Cas12a assisted tension sensor (MCATS) [159]. Copyright 2023, reproduced with permission from Springer Nature. (**b**) Overview of mechanotriggered hybridization chain reaction (mechano-HCR) based on TGT [160]. Copyright 2021, reproduced with permission from Wiley-VCH GmbH. (**c**) Principle of tension-activated cell tagging (TaCT). Stars represent Atto674N [161]. Copyright 2023, reproduced with permission from Springer Nature. (**d**) Schematic showing TGT-based rupture and delivery tension gauge system (RAD-TGT) [162]. Copyright 2023, reproduced with permission from Springer Nature. (**e**) Combination of DNA tensioners and microfluidic-based cell arrays. The DNA tensioner consists of three DNA sequences: F, Q, and H. Sequences H is divided into three parts: a (hybridizes with the cholesterol-labeled sequence F), b (assembled into a “hairpin” structure by the complementation of sequences b1 and b3), and c (hybridized with the quencher-labeled sequence Q) [163]. Copyright 2022, reproduced with permission from Wiley-VCH GmbH.

To convert tiny mechanical forces into easily quantifiable and amplified chemical signals, Ma et al. [158] proposed a TGT-based mechanically induced catalytic amplification reaction (MCR). This method can be used for high-throughput detection and readout of cellular surface receptor forces in the piconewton range. When cellular forces exerted through adhesion receptors cause TGT rupture, the anchored strand triggers MCR in situ, leading to the synthesis of long tandem repeats of DNA, which can be directly imaged through fluorescence in situ hybridization (FISH) or quantified using a high-throughput enzyme-linked immunoassay. Typically, the rupture of TGT are measured using inherently low-throughput and high-resolution methods. To achieve high-throughput readings, Duan et al. [159] developed an ultrasensitive detection method based on a mechano-Cas12a assisted tension sensor (MCATS), offering a simple and high-throughput measurement approach for platelet function evaluation and drug screening (Figure 6a). In MCATS, the activator for Cas12a is concealed by hybridization to a complementary strand and anchored to the substrate surface as double-stranded DNA. When cellular traction forces cause ssDNA rupture through ligands on the complementary strand, exposing the activator strand, efficient Cas12a nuclease activity is triggered, thereby mediating the cleavage of ssDNA reporter genes to produce abundant fluorescent signals detectable by an enzyme-linked immunoassay. Due to the sparsity of cellular mechanical events, molecular tension typically needs to be quantified using high-resolution fluorescence microscopy. To address this issue, Duan et al. [160] integrated mechanotriggered hybridization chain reaction (mechano-HCR) with TGT for detection and amplification of cell mechanics (Figure 6b). When the mechanical force of the cell membrane receptor–ligand exceeds the dsDNA force threshold, the dsDNA ruptures, exposing the anchored strand (initiator/primer) to trigger HCR in situ (with the addition of hairpin 1 and hairpin 2 for HCR amplification), thereby enhancing the signal-to-noise ratio of FMFS under the microscope and enabling the direct reading of cell receptor mechanical forces in a range of piconewtons using conventional enzyme linked immunoassays.

- Combination with Flow Cytometry

Ma et al. [161] proposed a tension-activated cell tagging (TaCT) to fluorescently label cells based on the magnitude of mechanical force exerted by cell receptors–ligands and utilized flow cytometry or FACS for the identification and sorting of mechanically active cells (Figure 6c). The TaCT probe consists of a load-bearing strand and a displacement strand, forming a DNA duplex. The load-bearing strand is modified with an RGD integrin–ligand at one end and attached to the substrate surface at the other end, internally coupled with a Cy3B dye. The complementary stripping chains are labeled with Atto647 and cholesterol. When the mechanical force F between the cell receptor and the ligand is less than the force threshold Fc of the TaCT probe, Cy3B and Atto647 form a FRET pair. When F exceeds Fc, the stripping chain labeled with Atto647 is released. It spontaneously inserts into the cell membrane of force-bearing cells through cholesterol, enabling high-throughput flow cytometry-based detection of active cells. The Cy3B dye on the load-bearing strand is quenched, enhancing fluorescence and thus visualizing the distribution of cellular force signals. To facilitate high-throughput mechanotype analysis, Pawlak et al. [162] proposed a TGT-based "rupture and delivery” tension gauge system (RAD-TGT). By combining flow cytometry and DNA sequencing techniques, they constructed a high-throughput analysis platform for analyzing cellular mechanical phenotypes based on the physical interactions between cells and their environment (Figure 6d). The anchoring chain in RAD-TGT contains a quencher at the 5’ end, and the ligand chain carries a fluorophore or a short nucleotide barcode at the 3’ end. When RAD-TGT ruptures under cellular traction forces exceeding the force threshold, its ligand chain will be internalized by the cell and will be detectable and recordable by flow cytometry (fluorescence signal) and DNA sequencing techniques (short nucleotide barcode), thus capturing the cellular mechanical force signals. To reveal the mechanical forces transmitted by individual receptor–ligands on nonplanar geometries, Hu et al. [164] developed a microparticle tension sensor (µTS) based on TGT for analyzing cellular mechanical forces on curved surfaces and for high-throughput flow cytometry readings. The FMFS are fixed on dispersible particles that are the size of cells. Cells exert mechanical forces through surface receptor–ligand bonds, causing the DNA duplex to rupture and produce a fluorescence signal. The generated fluorescence signals can be observed using high-resolution microscopy or read using high-throughput flow cytometry to quantify cellular molecular force signals.

- Combination with Microfluidic-Based Cell Array

Hang et al. [163] established a DNA tensioner platform based on a microfluidic device for high-throughput detection and piconewton-level resolution imaging (Figure 6e). The microfluidic chip within this platform utilizes a high-throughput micropore array, with each chip containing more than 10,000 micropores. DNA tensioners, which have a “hairpin structure” formed by self-assembly of three DNA strands, are affixed at the bottom of the micropores and can anchor to the cell membrane surface through hydrophobic interactions with cholesterol. The fluorescence intensity, which reflects the distance between the fluorophore and the quencher mediated by cellular forces, indicates the cellular mechanical force signals. This platform reported that the mechanical forces of drug-resistant tumor cells are higher than those of drug-sensitive tumor cells, as well as the mechanical differences between nonmetastatic and metastatic tumor cells. This platform provides new insights for complex mechanical studies, establishing connections between cellular mechanical heterogeneity and genetic heterogeneity.

#### 3.3.5. Cell–Cell Interaction

Mechanical forces between adjacent cells play a critical regulatory role in cellular function and communication. These intercellular mechanical forces are predominantly generated through interactions between cell membrane surface adhesion proteins (mainly cadherins) and their receptors. The development of methodologies to explore mechanical interactions between cells is of significant importance. Currently, the development of FMFS for measuring the mechanical forces between cells primarily focuses on the single-cell and collective-cell levels.

**Figure 7:**
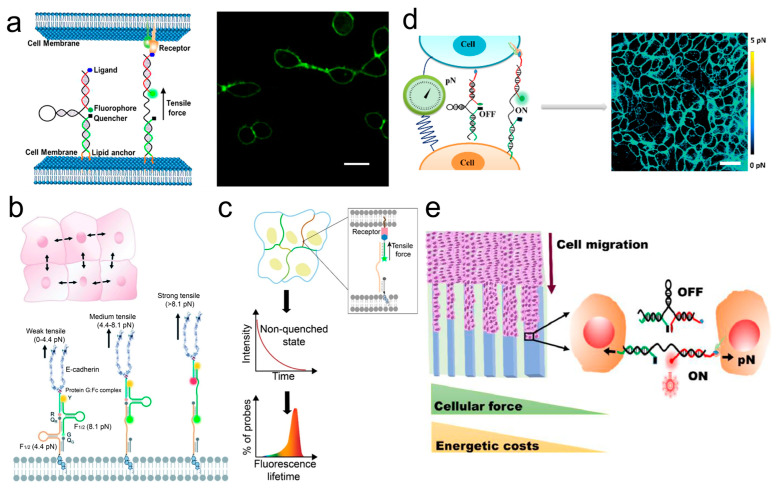
Examples of FMFS applications in cell–cell interactions. The arrows represent the direction of tensile forces generated by cells. (**a**) Schematic showing a membrane DNA tension probe (MDTP). Scale bars: 20 μm [165]. Copyright 2017, reproduced with permission from American Chemical Society. (**b**) Overview of a DNA-based ratiometric fluorescence probe (DNAMeter) [166]. Copyright 2020, reproduced with permission from the Royal Society of Chemistry. (**c**) Combination of DNA tension probe and fluorescence lifetime imaging microscopy (FLIM), named FLIM-MDTP [167]. Copyright 2021, reproduced with permission from Wiley-VCH GmbH. (**d**) Principle of a molecular tension fluorescence microscopy (MTFM) based on a “spring-like” DNA beacon. Scale bars: 25 μm [168]. Copyright 2020, reproduced with permission from American Chemical Society. (**e**) Intercellular forces and energy costs in confined microchannels [169]. Copyright 2023, reproduced with permission from Elsevier.

To detect mechanical forces between individual cells, Zhao et al. [165] reported a membrane DNA tension probe (MDTP) for visualizing the distribution of tensile forces at cell–cell junctions. They attached a pair of cholesterol molecules at one end of the MDTP, enabling the probe to spontaneously insert and anchor into the plasma membrane of live cells through hydrophobic interactions. When adjacent cells exert tensile forces on the ligands (located at the other end of the DNA hairpin) through transmembrane adhesion proteins (such as integrins and E-cadherins) that exceed the force threshold of the MDTP, probe unfolding results in a significant increase in fluorescence intensity. This enables the visualization of intercellular tensile forces (Figure 7a). Furthermore, MDTP can be used to study the effects of intercellular mechanical forces on collective cell behaviors. To broadly quantify intercellular mechanical forces in various collective cell behaviors and extend the measurement range of individual probes, Zhao et al. [166] developed a DNA-based ratiometric fluorescence probe named DNAMeter. The DNAMeter consists of two DNA hairpins with different force thresholds (force thresholds F_1/2_ of 4.4 pN and 8.1 pN ) and a lipid tail for anchoring on the plasma membrane of live cells. A reference fluorophore inside the DNAMeter normalizes its distribution on the membrane, and each DNA hairpin end is coupled with two orthogonal fluorophore–quencher pairs to report the magnitude of the mechanical force (Figure 7b). By measuring the ratio of each reporter gene to the reference fluorescence intensity, the distribution of cellular traction forces at cell–cell junctions can be visualized, quantifying these intercellular mechanical forces as weak (less than 4.4 pN), moderate (4.4–8.1 pN), or strong (greater than 8.1 pN) tensions.

For precise measurement of intercellular mechanical forces, Keshri et al. [167] reported a DNA tension probe based on fluorescence lifetime imaging microscopy (FLIM) named FLIM-MDTP. This probe is suitable for molecular tension imaging and quantification between cells and can simultaneously measure tensile forces between multiple ligand–receptor pairs (Figure 7c). By anchoring FLIM-MDTP to the cell membrane with the lipid portion, the mechanical tension between intercellular ligands and receptors induces the unfolding of the DNA hairpin and mediates the separation of fluorophore–quencher. This results in an increase in the number of probes in the unquenched state, manifesting as a longer fluorescence lifetime. To quantify the intercellular forces during collective cell migration in the wound healing process, Wang et al. [168] developed a MTFM method based on a “spring-like” DNA beacon. This approach utilizes the unfolding of the DNA spring to mediate changes in fluorescence intensity between fluorophores and quenchers as a reflection of intercellular mechanical forces. They discovered that high levels of intercellular mechanical forces and high energy costs exist at the wound edge (Figure 7d). Recently, Wang et al. [169] further investigated intercellular forces and energy costs in confined microchannels using the previously developed tension sensor (Figure 7e). They found that cells can adjust their intercellular forces to adapt to different confined environments, with greater intercellular forces and energy costs under highly confined conditions, resulting in more ordered cell orientations.

## 4. Conclusions and Perspectives

The dynamic interactions between cells and their microenvironment play a crucial role in various biological processes such as tissue regeneration, repair, and cell aging. The visualization and quantification of cellular forces during mechanotransduction are key to advancing our understanding of cellular mechanobiology. TFM and FMFS provide powerful tools for studying the intricate balance of cell-matrix interactions. This article reviews the imaging principles of TFM and FMFS, with a focus on the application of FMFS across a variety of biological processes.

TFM is one of the earliest tools used for measuring cell traction forces and has since been expanded to multidimensional measurements. By analyzing substrate deformation, TFM can determine the magnitude and direction of cellular forces, with the advantages of simpler operation and the capability to tune the substrate stiffness depending on the cell rigidity. However, because the sensitivity of TFM is coupled with the substrate stiffness, its spatial resolution and precision are low. In contrast, FMFS can be used to measure mechanical forces between cell membrane receptors and substrate ligands, and it can also measure in situ intracellular forces and interactions between cells. Based on the strain of the entropic polymer molecular springs, FMFS achieves single-molecule force resolution. Moreover, FMFS features high throughput and compatibility with a variety of materials, enabling the measurement of cellular forces on substrates such as glass, plastic, or hydrogels. However, FMFS can only provide the magnitude of cellular forces without offering directional information, and the design and preparation time are relatively long. Both TFM and FMFS can measure mechanical forces during interactions between cells and external ligands, allowing researchers to explore the mechanisms of cellular mechanotransduction and mechanical phenotype assessment in biological processes. Each method has its own advantages and limitations. To some extent, they can complement each other to visualize cellular traction forces simultaneously at subcellular and molecular scales [146].

At present, most FMFS are typically based on 2D models. Recently, Wang et al. [146] developed hydrogel-based FMFS to explore the process of stiffness-regulated taction forces transmission. This innovative work provides the potential for FMFS to measure cellular force directions and apply to 3D microenvironments. The FMFS used in 3D microenvironments will enable greater precision in the measurement of mechanical forces within subcellular structures in biological processes. This advancement may lead to the discovery of phenomena that have previously remained unobserved, such as the adhesive force of podosome protrusions in 3D hydrogels, and the mechanical forces of TCR–ligand interactions. Furthermore, this technique has great potential for applications in the imaging of cellular mechanical forces within internal structures of living tissues (such as tumors). However, the application of FMFS in 3D hydrogels faces many challenges such as fluorescent background noise and false force signals.

It is acknowledged that even under identical experimental conditions and measurement systems, differences may still occur between results obtained through different technologies. The Förster distance for typical FRET pairs is approximately 5 to 6 nm, and consequently, integrin–ligand forces that can be measured by FRET-based FMFS are limited by FRET efficiency, implying that cell forces that can be detected are not very large. In fact, in single-molecule FRET measurements using optical tweezers, the measurable force range is also on the order of 10 pN [122]. Nevertheless, by using titin-based nanoparticle tension sensors with clamped I27, Galior et al. [127] inferred that integrins tensions were up to 110 pN within focal adhesions. Despite the differences, measurements between FMFS and AFM have been reported to be close in some studies. For instance, through AFM measurement, the mechanical forces between integrins and several RGD-containing ligands in intact cells ranged from 32 to 97 pN [170]. Single-molecule force spectroscopy such as AFM and optical tweezers usually reveal bond strength through loading−rate−dependent rupture forces. The strength of integrin–ligand bond measured by AFM or optical tweezers depends on many factors, such as force−loading rates or the duration of force application, the type of ECM ligands used, and the nature of the integrin–ligand bond (catch bond and slip bond). For the same bond, varying the loading rate during measurement can lead to significant differences in the observed rupture forces [171,172]. For instance, when calibrating yellow fluorescent protein (YFP)-based FMFS, the unfolding force of the force-sensitive element YFP depends on the force-loading rate or the duration of force application [172]. The force sensitivity of FRET-based FMFS typically requires theoretical calibration through established polymer models or experimental calibration using single-molecule force spectroscopy techniques.

Integrin–ligand forces reported in different FMFS studies are not always consistent, which may be due to the differences between observation techniques, including force−loading rates adopted, the type of ECM ligands used, and the nature of the integrin–ligand bonds (catch bond and slip bond). The interactions between cells and external ligands are highly dynamic and transient, the molecular force-loading rate differing among various ligand–receptor pairs. Measurement of cellular force-loading rates provides possibilities for accurately monitoring mechanotransduction events in cells involving mechanical forces in the order of tens of piconewtons. Recently, Zhao et al. [150] developed an overstretching tension sensor (OTS) for determining single-molecule force-loading rates during the cell adhesion. OTS has great potential to reveal more about cell mechanics that could not be observed before.

Currently, many researchers are focused on combining FMFS with other methods (such as enzyme linked immunosorbent assays) to expand the application of FMFS. The construction of high-throughput detection platforms will provide a powerful mechanical tool for clinical cell detection and drug screening. In summary, FMFS is widely applied in the measurement of mechanical forces across a variety of biological processes, including the mechanical forces transmitted by intracellular proteins and the mechanical forces between cell–ECM interactions and cell–cell interactions, as well as the monitoring and regulation of cell behaviors. With the development of super−resolution technologies and the establishment of various platforms, FMFS has great applicational potential and will offer new insights into the mechanisms of cellular mechanotransduction.

## Data Availability

Not applicable.
